# Future Newborns with Opioid-Induced Neonatal Abstinence Syndrome (NAS) Could Be Assessed with the Genetic Addiction Risk Severity (GARS) Test and Potentially Treated Using Precision Amino-Acid Enkephalinase Inhibition Therapy (KB220) as a Frontline Modality Instead of Potent Opioids

**DOI:** 10.3390/jpm12122015

**Published:** 2022-12-06

**Authors:** Mauro Ceccanti, Kenneth Blum, Abdalla Bowirrat, Catherine A. Dennen, Eric R. Braverman, David Baron, Thomas Mclaughlin, John Giordano, Ashim Gupta, Bernard W. Downs, Debasis Bagchi, Debmalya Barh, Igor Elman, Panayotis K. Thanos, Rajendra D. Badgaiyan, Drew Edwards, Mark S. Gold

**Affiliations:** 1 Società Italiana per il Trattamento dell’Alcolismo e le sue Complicanze (SITAC), ASL Roma1, Sapienza University of Rome, 00185 Rome, Italy; 2 The Kenneth Blum Behavioral & Neurogenetic Institute, Austin, TX 78701, USA; 3 Division of Addiction Research & Education, Center for Mental Health & Sports, Exercise and Global Mental Health, Western University Health Sciences, Pomona, CA 91766, USA; 4 Institute of Psychology, ELTE Eötvös Loránd University, Egyetem tér 1-3, H-1053 Budapest, Hungary; 5 Department of Psychiatry, School of Medicine, University of Vermont, Burlington, VT 05405, USA; 6 Department of Psychiatry, Wright State University Boonshoft School of Medicine and Dayton VA Medical Centre, Dayton, OH 45324, USA; 7 Reward Deficiency Clinics of America, Austin, TX 78701, USA; 8 Center for Genomics and Applied Gene Technology, Institute of Integrative Omics and applied Biotechnology (IIOAB), Nonakuri, Purba Medinipur, West Bengal 721172, India; 9 Department of Precision Behavioral Management, Transplicegen Therapeutics, Inc., LLC., Austin, TX 78701, USA; 10 Department of Molecular Biology and Adelson School of Medicine, Ariel University, Ariel 40700, Israel; 11 Department of Family Medicine, Jefferson Health Northeast, Philadelphia, PA 19107, USA; 12 Ketamine Infusion Clinic of South Florida, Pompano Beach, FL 33062, USA; 13 Future Biologics, Lawrenceville, GA 30043, USA; 14 Department of Pharmaceutical Sciences, Southern University College of Pharmacy, Houston, TX 77004, USA; 15 Department of Genetics, Ecology and Evolution, Institute of Biological Sciences, Federal University of Minas Gerais, Belo Horizonte 31270-901, Brazil; 16 Center for Pain and the Brain (PAIN Group), Department of Anesthesiology, Critical Care & Pain Medicine, Boston Children’s Hospital, Harvard School of Medicine, Boston, MA 02115, USA; 17 Behavioral Neuropharmacology and Neuroimaging Laboratory, Clinical Research Institute on Addictions, Department of Pharmacology and Toxicology, Jacobs School of Medicine and Biomedical Sciences, University at Buffalo, Buffalo, NY 14203, USA; 18 Department of Psychiatry, South Texas Veteran Health Care System, Audie L. Murphy Memorial VA Hospital, Long School of Medicine, University of Texas Medical Center, San Antonio, TX 78229, USA; 19 Neurogenesis Project, Jacksonville, FL 32223, USA; 20 Department of Psychiatry, Washington University School of Medicine, St. Louis, MO 63110, USA

**Keywords:** neonatal abstinence syndrome (NAS), reward deficiency syndrome (RDS), genetic addiction risk severity (GARS), hypodopaminergia, dopamine homeostasis, opioids and alcohol common mechanism

## Abstract

In this nonsystematic review and opinion, including articles primarily selected from PubMed, we examine the pharmacological and nonpharmacological treatments of neonatal abstinence syndrome (NAS) in order to craft a reasonable opinion to help forge a paradigm shift in the treatment and prevention of primarily opioid-induced NAS. Newborns of individuals who use illicit and licit substances during pregnancy are at risk for withdrawal, also known as NAS. In the US, the reported prevalence of NAS has increased from 4.0 per 1000 hospital births in 2010 to 7.3 per 1000 hospital births in 2017, which is an 82% increase. The management of NAS is varied and involves a combination of nonpharmacologic and pharmacologic therapy. The preferred first-line pharmacological treatment for NAS is opioid therapy, specifically morphine, and the goal is the short-term improvement in NAS symptomatology. Nonpharmacological therapies are individualized and typically focus on general care measures, the newborn–parent/caregiver relationship, the environment, and feeding. When used appropriately, nonpharmacologic therapies can help newborns with NAS avoid or reduce the amount of pharmacologic therapy required and the length of hospitalization. In addition, genetic polymorphisms of the catechol-o-methyltransferase (COMT) and mu-opioid receptor (OPRM1) genes appear to affect the length of stay and the need for pharmacotherapy in newborns with prenatal opioid exposure. Therefore, based on this extensive literature and additional research, this team of coauthors suggests that, in the future, in addition to the current nonpharmacological therapies, patients with opioid-induced NAS should undergo genetic assessment (i.e., the genetic addiction risk severity (GARS) test), which can subsequently be used to guide DNA-directed precision amino-acid enkephalinase inhibition (KB220) therapy as a frontline modality instead of potent opioids.

## 1. Introduction

In this nonsystematic review and opinion, we primarily selected articles from PubMed to craft a reasonable opinion to help forge a paradigm shift in the treatment and prevention of primarily opioid-induced neonatal abstinence syndrome (NAS). Unfortunately, NAS from exposure to opioids in utero has reached epidemic levels worldwide. In infants, it is well known that nonpharmacologic modalities are the standard of care. However, pharmacotherapy is often required for the treatment of NAS. This article examines the current standard-of-care nonpharmacological and pharmacological treatments and explores additional potential alternative nonpharmacological treatments that could improve NAS treatment outcomes. 

### 1.1. Neonatal Abstinence Syndrome (NAS)

Newborns of individuals who use illicit and licit substances during pregnancy are at risk for withdrawal, also known as NAS. NAS is a spectrum of newborn neurobehavioral dysregulation symptoms that are complex, variable, and poorly understood. Although NAS is most frequently linked to opioid exposure, it can be related to other substances such as nicotine, methamphetamine, benzodiazepines, selective serotonin reuptake inhibitors (SSRIs), etc. [1,2]. The most common characteristic signs and symptoms of NAS indicate dysfunction in any of the following domains: motor and tone control, state control and attention, sensory integration, and autonomic functioning [3]. The onset of the signs and symptoms of NAS varies, depending on the history of substance exposure and the half-life of substance elimination. For opioids in particular, withdrawal can be delayed up to five days after birth or longer [4]. The presence and severity of these signs and symptoms provide the foundation for scoring systems (i.e., the Modified Finnegan Neonatal Abstinence Score, the Johns Hopkins Bayview Medical Center neonatal abstinence scoring sheet and initiation of treatment, etc.) that are used to make treatment decisions in newborns with NAS. The long-term consequences of NAS can include, but are not limited to, behavioral problems, neurodevelopmental delays, and when untreated, death [5].

### 1.2. Epidemiology and Economic Issues

Illicit drug use in the United States (US) has been steadily increasing and has reached unprecedented levels [6,7]. According to data from the National Survey on Drug Abuse and Health (NSDUH), in 2020 approximately 59.3 million individuals living in the US had used illicit drugs in the past year [6]. In addition, provisional data from the Centers for Disease Control and Prevention (CDC) revealed that there were approximately 107,622 drug overdose deaths in the US in 2021 [8]. This increase in illicit drug use in the US has resulted in an increase in NAS. In the US, the reported prevalence of NAS has increased from 4.0 per 1000 hospital births in 2010 to 7.3 per 1000 hospital births in 2017, which is an 82% increase [9]. 

In a study by Patrick et al. [10], birth and economic data from 580 counties over 7 years were reviewed. These included 1803 metropolitan country-years, 1268 rural country-years, and 927 remote county-years. An assessment inclusive of these years found 6,302,497 births with 47,224 births diagnosed with NAS. Specifically, the median NAS range was 7.1 per 1000 hospital births. Notably, individuals diagnosed with NAS are associated with the 10-year unemployment rate. Victims of NAS in this global investigation presented in the lowest unemployment quartile. 

## 2. Treatment of NAS

The management of NAS is varied and involves a combination of nonpharmacologic and pharmacologic therapy. The treatment goals for NAS include preventing NAS-associated complications and restoring normal newborn activities, such as nutrition intake, weight gain, sleep, and adjustment to the social environment [11]. As with all pharmacological treatments, the potential risks and benefits of treatment must be considered for each patient. For example, some disadvantages include prolonged hospitalizations and drug exposure, while some advantages include the relief of withdrawal symptoms and the prevention of potential complications.

### 2.1. Nonpharmacological Interventions 

Nonpharmacologic care should be initiated at birth for all substance-exposed newborns, and the parent/caregiver should also be actively involved. It should continue throughout the newborn’s hospitalization and after discharge, regardless of the need for pharmacologic treatment. When used appropriately, nonpharmacologic therapies can help newborns with NAS avoid or reduce the amount of pharmacologic therapy required. However, they do not serve as a substitute for pharmacotherapy when it is necessary. Nonpharmacologic therapies entail individualized assessments of the newborn and parent/caregiver’s functioning; the environment, to determine specific newborn–parent/caregiver dyad triggers for dysregulation; and adaptative responses to the environment to reduce physiological and neurobehavioral symptoms and promote newborn–parent/caregiver dyadic regulation [3].

*General care measures* [3,12]:

General care measures in NAS include identifying the signs, symptoms, and triggers of physiological behavioral dysregulation and individualizing the care of the newborn based on these observations as well as promoting organization, competence, and physiological stability in newborns by identifying techniques that improve symptomatology that are specific to each newborn. For example, gentle vertical rocking can help reduce excessive irritability. Moreover, tremors and hypertonicity can be reduced by utilizing swaddling and positioning (i.e., the side-lying C position), which decrease motoric hyperactivity and allow newborns to organize their behaviors and become calm. 

*Newborn–parent/caregiver relationship* [3]:

Nonpharmacologic interventions in this domain include assessing parental functioning and interaction with the newborn to help reduce dysregulation and promote dyadic synchronization [13]; educating the parent/caregiver on how to identify the signs of withdrawal; teaching the parent/caregiver about their newborn’s sensitivities; helping the parent/caregiver develop strategies and respond to the newborn in a manner that reduces the newborn’s dysregulation and expression of NAS; aiding the parent/caregiver in understanding their feelings surrounding their newborn’s functioning so they can respond more appropriately; and managing maternal issues such as mental illness, limited health care access, intimate partner violence, etc., in order to maintain a healthy newborn–parent/caregiver relationship, which is vital for the newborn’s development [5]. 

*Environment* [12]: 

Nonpharmacologic interventions in this domain include identifying potential sensory and environmental input sources of dysregulation for newborns and altering the environment to minimize their effects and dysregulation. For example, a newborn who becomes hypertonic or irritable with eye contact might need the parent/caregiver to avoid eye contact while feeding, handling, or performing other activities together. In addition, a newborn who becomes easily overstimulated by noise can be cared for in a quiet area. Finally, rooming-in (i.e., the colocation of the newborn and the parent/caregiver after delivery and beyond) has been found to reduce NAS severity [12,14,15] and is recommended in the inpatient setting. 

*Feeding*:

For newborns with NAS, formula feeding should not necessarily be the default. In fact, breastfeeding has been shown to be successful in some individuals with opioid use disorder (OUD) [12,16,17,18]. Recommendations involving an individual’s suitability for breastfeeding should be tailored for individuals with one or more of the following traits: the concurrent usage of other prescription medications; participation in prenatal care and/or substance use disorder (SUD) treatment during or after the second trimester; and relapse during the third trimester with abstinence maintained for 30 days prior to delivery. 

Breastfeeding by methadone-maintained individuals seems to be safe and can lessen the severity of NAS and the necessity for pharmacological intervention [19,20,21,22]. The concentrations of methadone have been found to be low in human breast milk (range: 21–462 ng/mL) and do not appear to be associated with the parent’s methadone dose [19]. The low concentrations of methadone found in human breast milk are unlikely to have a significant impact on the newborn’s display of NAS, and other breastfeeding-related variables could be responsible for the decreased severity of NAS in breastfed infants of methadone-maintained individuals. In addition, buprenorphine is excreted in low concentrations into human breast milk and seems to be safe for newborns of buprenorphine-maintained individuals [23,24].

### 2.2. Pharmacological Interventions

Pharmacological management is initiated for newborns who display significant signs and symptoms of NAS despite adequate and personalized nonpharmacological care. The goal of pharmacological management is a short-term improvement in NAS symptomatology. Currently, opioid therapy is the preferred first-line treatment for NAS. This is based on limited data that show opioid therapy reduces the need for additional medications and shortens hospital stays [5,12,25,26,27,28]. Morphine and methadone are the preferred opioids, and the selection is based on the clinician/hospital. Morphine is typically the preferred agent of the two, while methadone is considered a reasonable alternative. However, there have been studies that indicate that methadone minimally reduces hospital stay and treatment duration when compared to morphine [29,30]. Buprenorphine is another agent that has been used and appears to be effective in the treatment of NAS [31,32]. However, its use in newborns is limited due to the high ethanol content (30%) and its challenging sublingual administration [12].

A 2020 systematic review by Zankl et al. identified 16 trials including 1110 infants [5]. In one of the trials (N = 80 infants), morphine was compared to supportive care alone, and the results showed that morphine increased the length of treatment and hospitalization but shortened the time needed to regain birthweight. In trials that compared morphine to methadone (two trials, N = 147 infants), it was found that they both had comparable rates of breastfeeding success, length of hospitalization, and treatment failure. In trials that compared morphine to buprenorphine (three trials, N = 113 infants), it was found that they both had comparable rates of treatment failure, but the length of hospitalization was shorter in the buprenorphine group. In a separate network meta-analysis utilizing both indirect and direct comparisons (18 trials, N = 1072 infants), six medications were evaluated, including morphine, methadone, buprenorphine, clonidine, phenobarbital, and DTO. Morphine and methadone were associated with the lowest rates of treatment failure, but the differences were not statistically significant [33]. Additionally, buprenorphine was found to have the shortest length of hospitalization. 

In addition, according to a meta-analysis by Cleary et al., there were no statistically significant differences in the incidence of NAS in newborns of women on higher doses of opioids when compared to lower doses in studies that used an objective NAS scoring system and prospective studies [34]. Similarly, Bakstad et al. reported that the maternal methadone or buprenorphine dose was not predictive of the occurrence or need for NAS treatment in newborns [35]. 

A second medication is sometimes required in newborns who have severe NAS that is not sufficiently controlled with a single agent [5,33,36,37]. The two most commonly used second-line medications are clonidine and phenobarbital. Typically, clonidine is the preferred second-line medication due to concerns regarding phenobarbital’s adverse effects, including oversedation, a high alcohol content, challenges with weaning substance-exposed newborns from phenobarbital, and phenobarbital’s potential long-term impacts on neurodevelopment based on animal studies [12,38,39,40,41]. In addition, the concurrent use of phenobarbital and clonidine appears to reduce the consequences of opioid-induced negative neuronal development in newborns with NAS [36,42].

Agthe et al. found that, in a clinical trial (N = 80) with newborns who had intrauterine exposure to heroin or methadone, the addition of clonidine to standard opioid therapy was found to decrease pharmacological treatment (11 vs. 15 days) when compared to placebo [43]. The placebo group also required higher dosages of opioid therapy. No significant short-term complications, such as bradycardia, hypotension, hypertension, or oxygen desaturations, were observed in either group. However, the clonidine group had three deaths (sudden infant death syndrome (SIDS), myocarditis, and homicide). Johnson et al. compared the use of phenobarbital and opioid therapy together to opioid therapy alone and found that the combined therapy decreased the length of hospitalization and the duration of symptoms when compared to opioid therapy alone [44]. However, despite the use of phenobarbital in the treatment of NAS, no safety profile has been established, and the alcohol content remains a concern [45]. Finally, in a retrospective multicenter study (N = 563) by Merhar et al., it was found that the length of hospitalization and morphine treatment was shorter for newborns who were treated with a combined therapy of morphine and phenobarbital compared to those who were treated with a combined therapy of clonidine and morphine [37]. However, more newborns were discharged on phenobarbital than clonidine (78% vs. 29%).

### 2.3. Neurodevelopmental Issues with Opioid Treatment

Czynski et al. [46] reported that the prevalence of NAS has increased by 333% over the last two decades, which translates to approximately one infant born every 15 min in the United States [47]. This unfortunate statistic reveals that 50–80% of newborn infants exposed to opioids in utero develop NAS. Along these lines, Boardman et al. [48] suggested that a literature summary of 40 years necessitated a reassessment of ways to treat NAS without opioids, even during withdrawal periods. These investigators identified knowledge gaps and urged the scientific community to re-evaluate childhood clinical outcomes such as infant brain development and visual and long-term neurocognitive function. Van den Hoogen et al. [49], assessing the behavioral and cFos responses, known to be a marker for neuronal activation in neonatal animals withdrawing from opioids, found increased cFos expression in spinally projecting neurons within the periaqueductal grey (PAG), locus coeruleus, and rostral ventromedial medulla (RVM). They also observed that the narcotic antagonist naloxone precipitated profound withdrawal symptoms across all developmental levels and stages within several key brainstem regions. Another example of neurodevelopmental issues linked to opioids was investigated by others [46], involving mothers maintained on methadone or buprenorphine but randomized to morphine vs. methadone. Czynski et al. [46] reported that adding phenobarbital to the treatment routine resulted in several medical problems, suggesting that sedative hypnotics may not be an appropriate modality in these NAS cases. Finally, Witt et al. [50] evaluated long-term childhood and infant mortality involving 1900 individuals diagnosed with NAS and 12,283 controls. The results indicated that NAS-diagnosed children were readmitted to the hospital within five years of life more frequently when compared to non-NAS controls. Most perplexing was the finding that in NAS patients there was an unadjusted significant increased mortality risk ratio of 1.94 (95% CI 0.99–3.80). Witt and associates [50] concluded that childhood readmission due to NAS “argues” for innovative (possibly nonpharmacological) early interventions to prevent morbidity and possibly mortality. 

### 2.4. A Case in Favor of Non-Opioid Treatment in NAS

Opioid pharmacokinetics are influenced in neonates by a higher body water content that can alter drug distribution and metabolic processes that are not mature and can lead to low plasma protein and liver enzyme activity. For example, these factors could affect cleared metabolites, resulting in a prolonged half-life of opioids. This is further complicated by reduced renal excretion, which might be due to immature tubular secretion, glomerular filtration, and reabsorption [51]. In addition, animal experiments have shown that an immature blood–brain barrier in neonates may result in an augmented sensitivity to opioids [52].

An argument against the utilization of opioids to treat NAS has been espoused by some investigators globally [53,54,55,56], and a word search for “acupuncture and NAS” revealed 102 PubMed listings (26 January 2022). Following an extensive review of the literature, including the Cochrane Databases, Jackson et al. [53] reported that acupuncture is a safe and effective nonpharmacological alternative to potent opioids for the treatment of NAS. 

Similarly, while not in neonates but in adults, our laboratory observed a significant attenuation of opioid withdrawal symptoms with a well-researched nutraceutical complex prodopaminergic regulator (KB220Z) as an aqua-power liquid variant [57]. Out of 17 heavily opioid-dependent patients during detoxification, only three received buprenorphine/naloxone (Bup/Nx) along with KB220Z. We first used a dose of KB220Z of two ounces (oz) twice daily before meals, along with other detoxifying agents, including clonidine, benzodiazepines, and gabapentin. The dose of KB220Z was maintained for six days in five patients. Then, in a second scenario, we employed a higher dose of four oz every six hours over six days. The higher dose was utilized in another 12 patients. Only three people relapsed with these two protocols during the first two weeks of the experiment. Importantly, the remaining 82% were maintained on KB220Z. Specifically, these subjects were maintained without any additional Bup/Nx for a minimum of 120 days and, in one subject, 214 days. One limitation is the inability to interpret these results or make any conclusions regarding specific KB220Z effects.

If further confirmed in more extensive studies, using KB220Z for opiate/opioid detoxification may provide a novel way to eliminate the need for addictive opioids during withdrawal and detoxification. This paradigm shift, which requires extensive research, may translate to a reduction in universally employing powerful and addictive opioids to treat OUD and NAS [58].

### 2.5. Snapshot of Dopaminergic Mechanisms in Addiction

**Figure 1:**
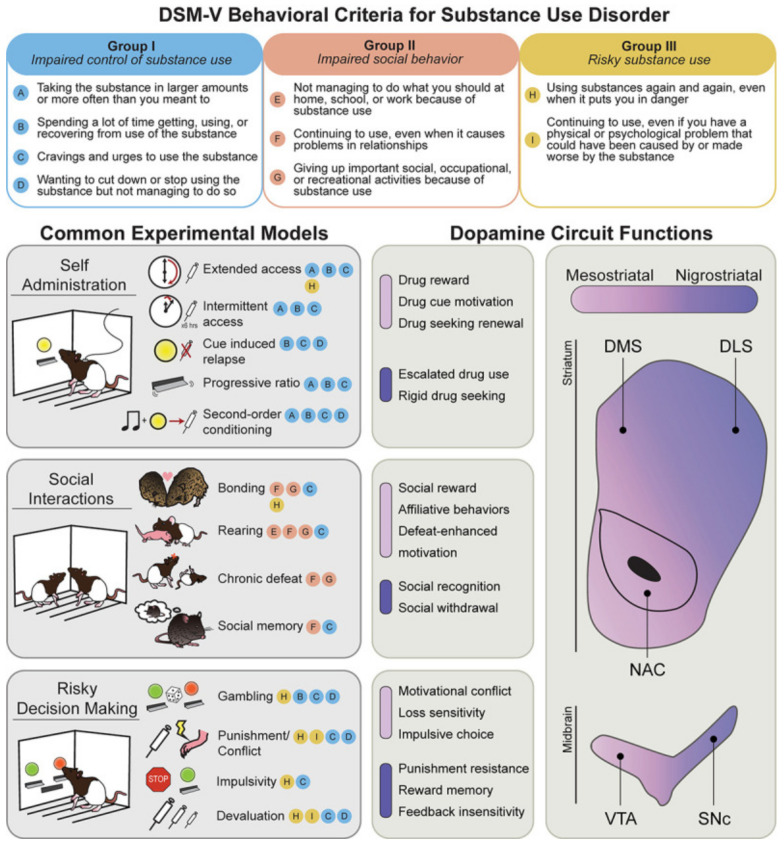
Behavioral models are used to classify the phenotypes of substance use disorder (SUD). (**Top**) The behavioral criteria of SUDs (circled letters) can be sorted into three main categories: impaired control of substance use (Group I), impaired social behavior (Group II), and risky substance use (Group III). (**Left**) Common rodent experimental models and the SUD criteria that are thought to best approximate them. Note that most models capture multiple SUD features. (**Right**) Mesostriatal circuits (light purple), including dopamine projections from the ventral tegmental area (VTA) to the nucleus accumbens (NAc), and nigrostriatal circuits (dark purple), including dopamine projections to the dorsomedial (DMS) and dorsolateral striatum (DLS), generally have dissociable roles in different components of major SUD models. The middle panels list the most clearly defined roles for these two systems in each SUD category.

Undoubtedly, substance and nonsubstance behavioral addiction is a complex genetic and epigenetic disease that afflicts millions worldwide. Clinically, a major issue biologically is a breakdown in the function of the brain’s reward circuitry in many cases, even in newborns, especially as a function of specific known genetic risk variants as antecedents compounded by the epigenetic effects of in utero opioids that impact behavior. It is of interest that much of our knowledge of the neurobiological underpinnings of human behavior regarding “reward dysfunction” has been derived from animal research. Poisson et al. [59] recently reviewed the evidence related to the critical role of striatal dopamine (DA) in all addictive behaviors. While our laboratory has been at the forefront of DA genetics and its association with not only severe alcoholism but also general reward deficiency [60,61], Poisson and associates [59] contributed to the identification of specific and distinct mesostriatal and nigrostriatal DA circuit functions in substance use disorder (SUD). Although at least seven major neurotransmitter systems (serotoninergic, endorphinergic, GABAergic, glutamatergic, opioidergic, acetylcholinergic, and dopaminergic) are involved, striatal dopamine is essential for controlling one’s craving behaviors, impairments in decision making that underlie several risk-taking behaviors, anti-socialization, and overall compulsive and impulsive behaviors (Figure 1).

As Poisson et al. [59] pointed out, most of the brain’s dopamine neurons are in two midbrain regions (Figure 1): the ventral tegmental area (VTA) and the substantia nigra pars compacta (SNc). Others revealed that DA neurons in the VTA mainly project to the ventral striatum, specifically the nucleus accumbens (NAc) core and shell. The NAc shell comprises the mesostriatal pathway and links to certain frontal regions in the prefrontal cortex, pallidum, and amygdala [62,63]. The work of Nestler’s group [64] and others [65,66] has shown that DA neurons in the VTA intermingle with GABAergic and glutamatergic neurons. In contrast, the SNc DA neurons project to the dorsomedial (DMS) and dorsolateral (DLS) striatum almost exclusively and comprise a well-known nigrostriatal system [67,68,69]. Importantly, in the striatum, Gerfen [70] showed that DA neurons contact GABAergic medium spiny neurons (MSNs) that contain excitatory type 1 (D1-MSNs) or inhibitory type 2 (D2-MSNs) DA receptors. Kupchik et al. [71] have further confirmed this work. The primary role of DA’s modulatory effect on striatal activity due to these outputs involves the control of specific behaviors (such as motivation and reward learning). It is indeed well known that most highly addictive psychoactive drugs (such as cocaine, alcohol, and morphine) cause the release of DA in the NAc and other striatal regions. According to Collins and Saunders [72], based on terminal mechanisms, DA release may play an essential role in many infractions related to aberrant drug use and cravings and even drug reinstatement or relapse, the cornerstone of unwanted SUD [73,74,75,76,77]. A review of the literature revealed that DA neurons across the VTA and SNc circuitry impact a wide array of behavioral functions, showing significant overlap or co-occurrence across many reward-related behaviors [78,79,80,81]. Mesostriatal DA neurons contribute to the execution of goal-directed behaviors and learning. However, nigrostriatal DA, specifically in the DLS, impacts movement control and even the execution of rigid habitual actions that translate to addiction heterogeneity [82,83,84,85,86,87]. It is important to recognize that DA has a powerful effect on many behaviors that, when impaired, induce in the reward circuitry maladapted dysfunctional behaviors and addiction, including poor decision making, a prominent underpinning of compulsive behaviors [88,89,90,91,92,93,94,95,96,97,98,99,100,101,102,103,104,105,106,107,108,109,110,111,112,113,114,115,116,117,118,119].

### 2.6. Evidence-Based Prodopaminergic Regulation (KB220)

Genetic polymorphisms of the catechol-o-methyltransferase (COMT) and mu-opioid receptor (OPRM1) genes appear to affect the length of stay and the need for pharmacotherapy in newborns with prenatal opioid exposure [120]. These findings are consistent with data from adult studies that demonstrated that variations in these genes are associated with adult opioid dependence variability [121]. Epigenetic modifications to the OPRM1 gene have also been linked to the severity of NAS [122]. Thus, it appears prudent to incorporate genetic testing in order to reveal reward circuitry gene polymorphisms, especially those associated with dopaminergic pathways and opioid receptors, as a means of improving NAS treatment outcomes [123].

Therefore, after a decade of attempting to reduce severe NAS symptomatology with nonpharmacological approaches, including acupuncture and transcranial stimulation [124,125], we propose adding the complex prodopaminergic regulator (KB220Z) based on genetic assessment (i.e., the genetic addiction risk severity (GARS) test), along with other non-opioid modalities. KB220Z is a formulation of enkephalin, enkephalinase inhibitors, and dopamine-releasing neuronutrients that is utilized to induce dopamine homeostasis for the detoxification and treatment of individuals genetically predisposed to developing addictive and compulsive behaviors known as reward deficiency syndrome (RDS) (Table 1) [61]. The formulations are based on the results of the GARS test, which evaluates the presence of reward genes and risk alleles and can successfully stratify the potential for developing OUD-related risks (Table 1) [61]. In addition, second-line non-opioid pharmacological agents such as clonidine [126] could also be used in the short term, followed by longer-term dopamine regulation with KB220 variants. 

In the most recent reiteration, additional nutrients have been added to the formula, such as *β*-nicotinamide adenine dinucleotide (β-NAD) to function as a catalyst for dopamine synthesis [127] and N-acetyl-cysteine [128] to help promote glutaminergic drive in the VTA to release DA in the NAc. 

It is noteworthy that globally we are facing a significant challenge in the increased utilization of opioids to reduce ongoing stress. In 2021, the USA had over 100,000 narcotic-overdose-induced fatalities [129]. Certainly, NIAAA and NIDA continue to struggle with innovation to help reduce or eliminate this catastrophic epidemic. However, we are concerned with the current FDA-approved medication assistance treatments (MAT). The concern is that MAT works by blocking dopamine function and release at the preneuron in the NAc. While we understand short-term use to reduce harm [130], we oppose long-term use for tertiary treatment [131]. 

The research-based neuronutrient KB220, which has been intensely investigated in at least 38 studies, has demonstrated clinical benefit [45]. The effects include, but are not limited to, reduced against medical advice (AMA) rates; reward system activation, including blood oxygen level dependent (BOLD) dopamine signaling; a reduction in craving behavior; relapse prevention; and the attenuation of stress, anger, and aggressive behaviors. It is noteworthy that our group has recently published an additional twelve studies utilizing KB220 variants [132]. Based on animal research and clinical trials, the prodopamine regulator KB220 shows promise in treating addiction and pain as well as opioid-induced withdrawal symptomatology [133]. 

While additional neurobiological and genetic studies are required to help understand the mechanism of action of this neuronutrient, possible studies related to NAS seem prudent. However, the evidence advocates for the induction of “dopamine homeostasis” [105]. We believe that the utilization of this nonpharmacological approach could enable an approach free from side effects that induces the “normalization” of brain neurotransmitter signaling epigenetically. Moreover, it is reasonable to predict that the utilization of this neuronutrient (KB220) could lead to improved function and the attenuation of NAS. 

It is vital that addiction researchers realize that providing opioids to treat opioid abuse is counterproductive and lacks ways to induce real potential prophylaxis. Instead, we propose long-term prophylaxis utilizing our concept of coupling GARS to help determine precision prodopaminergic regulation via KB220. 

With that said, we are encouraged by these results, as published over the last 50 years. We look forward to continued advancements in appropriate nutrigenomic solutions for the millions of victims of all addictions, including reward surfeit syndrome (RSS) in adolescents and RDS in adulthood [105] as well as addictions to substances such as drugs and food and to behaviors such as smoking, gambling, and gaming, especially in our next generation. Understanding these simple precepts may engender novel ways to treat NAS with nonpowerful opioids.

**Figure 2:**
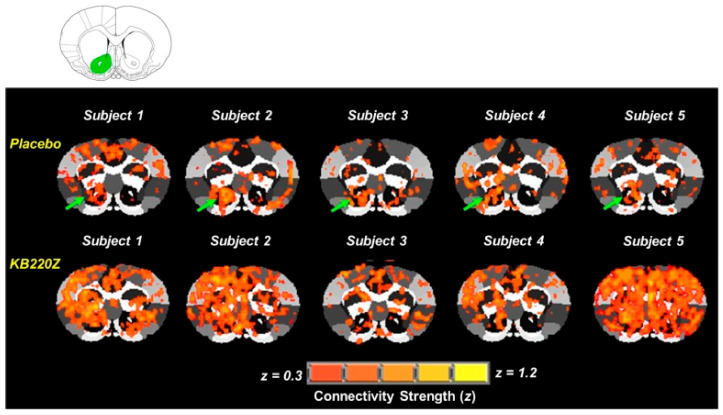
Representative cross-correlation maps show five subjects: placebo-controlled and KB220Z-treated rats. The maps correspond to the resting-state connectivity for the NAc (highlighted in green in the atlas map above the figure; only the left seed is shown). Note the distributed but significant connectivity between various brain regions and the NAc in the placebo subjects. KB220Z increased connectivity, especially between the left and right accumbens, dorsal striatum, and limbic cortical areas, such as the anterior cingulate, prelimbic, and infralimbic regions. Correlation maps for the representative subjects are presented at a threshold of 0.3 ≤ *z* ≥ 1.2 [135] (with permission).

The neurological effects of KB220 in naïve rodents, uncovered in studies conducted by Marcelo Febo [134], showed BOLD activation using KB220 in regions of interest related to the brain reward circuitry. Specifically, there was a significant increase in the functional connectivity of the NAc with the medial and lateral anterior thalamic nucleus and the surrounding somatosensory cortex (Figure 2). Another important finding revealed that KB220Z augmented the connectivity between corticothalamic areas and this region of the reward system.

Additionally, with KB220Z vs. placebo, when the anterior thalamic nucleus was the selected seed RIO, there was minimal evidence of connectivity observed outside this area. Febo et al. [134] found a significant enhancement in connectivity with surrounding sensory cortical areas and the regions mentioned above, including the NAc (both left and right). A more substantial effect on the resting state functional connectivity (rsFC) in the dorsal hippocampus was of real interest. Furthermore, connectivity was increased between the left and right dorsal hippocampi, the upper limb somatosensory regions, the NAc and limbic cortical areas, and the anterior cingulate (Figure 2).

A follow-up study utilizing KB220Z was also administered to abstinent Chinese heroin abusers to help map the brain reward circuitry interaction potential in humans. Along these lines, it is noteworthy that Willuhn et al. [136] reported that cocaine use and even non-substance-related addictive behavior surge as dopaminergic function is decreased. Understanding that reduced or deficient levels of brain DA enhance heroin-seeking behavior. Treatment strategies, including a DA agonist therapy that conserves dopamine function, could prevent relapse to opioids. 

The effect of KB220Z on the reward circuitry of ten heroin addicts undergoing protracted abstinence for an average of 16.9 months was investigated [135]. Specifically, in a randomized, placebo-controlled crossover study of KB220Z, five subjects completed the triple-blinded experiment. Additionally, nine patients were genotyped utilizing the GARS test. 

KB220Z induced an enhanced BOLD activation in caudate–accumbens–dopaminergic pathways compared to placebo following a one-hour acute administration. Moreover, KB220Z also attenuated the resting state activity in the putamen of abstinent heroin-dependent subjects. In the second phase of this preliminary investigation of all ten abstinent heroin-dependent patients, three brain regions of interest were significantly activated from the resting state by KB220Z compared to placebo.

Interestingly, augmented functional connectivity was observed in a putative network that included the cerebellum, medial frontal gyrus, dorsal anterior cingulate, NAc, occipital cortical area, and posterior cingulate. These results and other qEEG studies [137,138] support the notion of a putative anticraving/anti-relapse role for KB220Z in opioid dependence by direct or indirect dopaminergic interaction. 

Preclinical experiments and human trials associated with KB220 variants have been published and reviewed [45]. Early formulations of KB220 increased brain enkephalin levels in rodents [136], reduced alcohol-seeking behavior in C57/BL mice [135], and converted ethanol-preferring C57/BL mice via pharmacogenetics to the same level of nonpreference as alcohol-averse DBA mice [136]. Thus, based on these and other animal and human studies [137,138,139,140,141,142,143,144,145,146,147,148,149,150,151,152,153,154,155,156,157,158,159,160,161,162,163,164,165,166,167,168,169], using KB220Z might be an ideal treatment for NAS, particularly to counteract underlying brain hypodopaminergia.

### 2.7. Common Neurochemical Mechanisms Related to Alcohol and Opiate/Opioid-Induced Withdrawal Symptomatology

Wallace et al. [170] reported that as many as 47% of pregnant women misuse/abuse alcohol, and at least 6% misuse or abuse illegal drugs such as opioids. The European Monitoring Centre for Drugs and Drug Addiction has noted that approximately 500 thousand opioid-dependent Europeans are, unfortunately, on opioid maintenance substitution therapy (OMST) [171]. It is indeed a fact that about 30,000 opioid-dependent women have become pregnant [172]. The treatment of women involved with a combination of alcohol and opioid dependence is very complex and is a challenge that must be faced to attenuate the onslaught of unwanted NAS [173,174]. 

Since the early 1970s, Blum’s group has investigated the common neurochemical and genetic underpinnings of all addictive behaviors. One area of investigation by this group was a common mechanism among opiates, alcohol, and neurotransmitter involvement in withdrawal symptomology—the commonality concept related to condensation products derived from the identification of in vivo isoquinoline formation. There is enough evidence to suggest that these condensation amines “link” to opiates. The message here is that when one imbibes alcohol, opiate-like isoquinolines are formed [175]. These isoquinolines induce a robust enhancement of ethanol-induced withdrawal symptoms (EIW) [176]. For example, a series of experiments revealed that the inhibition of catecholamine synthesis results in the potentiation of EIW [177]; haloperidol, a D2 dopamine receptor (DRD2), potentiates EIW [178]; serotonergic blockers potentiate EIW [179]; dopamine suppresses EIW [180]; morphine suppresses EIW [181,182]; naloxone inhibits alcohol dependence [183]; and clonidine enhances EIW [184,185,186].

Of interest is the finding that by employing quantitative electroencephalography (qEEG) as an imaging tool, Miller et al. [153] showed the impact of one formulation of KB220 as a putative activator of the mesolimbic system. These investigators [153] found that intravenous administration reduces or “normalizes” aberrant electrophysiological parameters of the brain reward circuitry region. Specifically, KB220 significantly normalized widespread theta and alpha activity in alcoholics and heroin abusers, showing several neurotransmitter-linked polymorphic genes measured by the GARS test. The authors [153] suggested that the chronic activation of dopaminergic receptors, such as DRD2, will increase upregulation, induce an augmented “dopamine sensitivity,” and ultimately “enhance the sense of happiness,” specifically, for example, in carriers of the DRD2 A1 allele. 

### 2.8. Genetic Addiction Risk Severity (GARS): Promoting the Early Identification of Polymorphic Risk Alleles in RDS

Since 1990, when our laboratory published the DRD2 Taq A1 allele and severe alcoholism association in JAMA, there has been an explosion of genetic candidate association studies, including genome-wide association studies (GWAS). To develop an accurate test to help identify those at risk for at least alcohol use disorder (AUD), Blum’s group developed the GARS test, consisting of ten genes and eleven associated risk alleles. To statistically validate the selection of the risk alleles measured by GARS for alcohol, we applied a strict analysis to studies that investigated the association of each polymorphism with AUD or AUD-related conditions published from 1990 until 2021. This analysis calculated the Hardy–Weinberg equilibrium of each polymorphism in cases and controls. Pearson’s χ2 test or Fisher’s exact test were applied to compare the gender, genotype, and allele distribution, if available. The statistical analyses found the 95% CI for the odds ratio (OR) and the post hoc risk for alcoholism prevalence to be an estimated 8% of the population, revealing a significant detection. The likelihood ratio (LR) results also showed significance for DRD2, DRD3, DRD4, dopamine transporter gene (DAT1), catechol-o-methyltransferase (COMT), mu opioid receptor gene (OPRM1), and serotonin transporter gene (5-HTT) at 5%. The United States and European patents on a ten-gene panel and eleven risk alleles had been issued prior to this statistical analysis. 

One possible etiological root cause of addiction involves the identified neurotransmitter network function within the mesolimbic and prefrontal cortex (PFC) brain regions. It is essential that the scientific community recognizes that the subsequent regulation of the final reward and motivational pathway of “wanting” translates to the physiological induction of “normal” neuronal dopamine release. The typical neuromodulating aspects of neurotransmission and its disruption from chronic exposure to drugs and behavioral addictions necessitate an approach that involves achieving “dopamine homeostasis,” especially for AUD and other unwanted RDS behaviors [187,188,189,190]. Interestingly, Bidwell et al. [191], utilizing functional MRI imaging, found that during alcohol cueing DRD2 promoter methylation was strongly associated with responses to alcohol cues in many brain circuitry regions related to reward. In addition, the clinical metrics of AUD severity were positively associated with methylation of the promotor region of the DRD2 gene. This finding details early work from our laboratory [60], whereby we suggested that the DRD2 A1 allele residing outside of the promotor region in the 3′ region of the genome, now known to be involved in the transcription process related to mRNA expression, is the root cause of severe alcoholism. 

**Figure 3:**
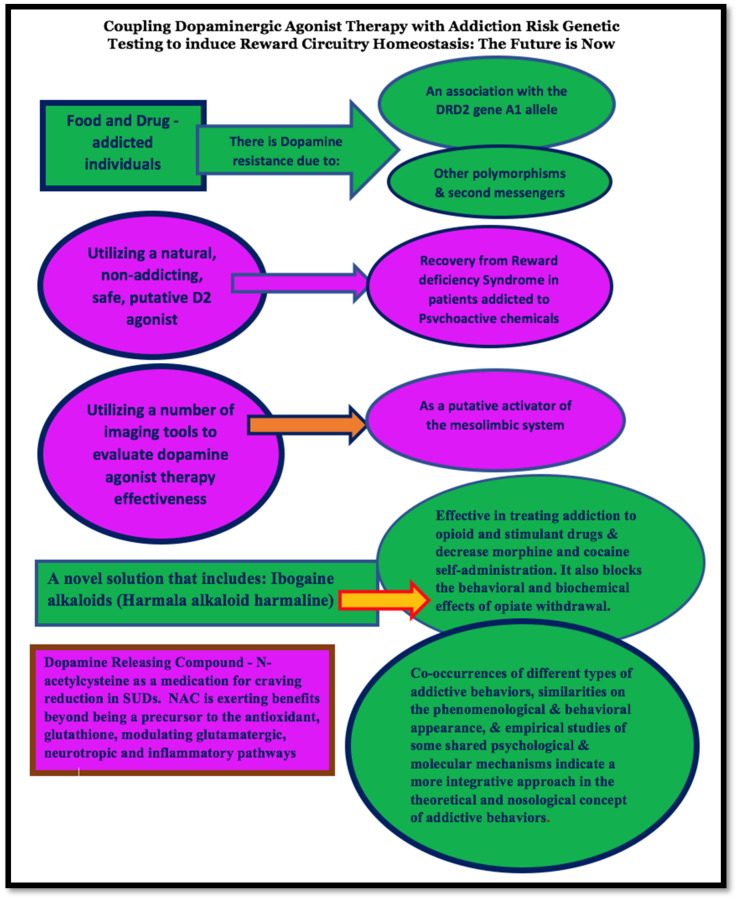
Schematic of our proposed model to treat and identify genetic antecedents and to provide a way of inducing “dopamine homeostasis.”

Figure 3 displays this article’s primary tenant. It provides a schematic showing the interactive events related to coupling gentle dopaminergic agonists (not potent D2 agonists such as bromocriptine) with genetic risk testing as one way to induce homeostasis of the brain reward circuitry. 

Blum’s laboratory has worked to successfully develop the GARS test, an accurate genetic test to predict risk liability for RDS behaviors, including AUD. The association to determine risk using a clinical outcome, the addiction severity index media version (ASI-MV), was accomplished with the Institute of Behavioral Genetics, University of Colorado, Boulder. Ten reward candidate genes were selected to develop this patented GARS test. They included the dopamine receptors (DRD1, 2, 3, and 4); DAT1; 5-HTT, COMT, monoamine oxidase (MAO), gamma-aminobutyric acid (GABA), OPRM1, and some single nucleotide polymorphisms (SNPs) and point mutations, all chosen to reflect a hypodopaminergic trait. The functions of the chosen alleles of the ten genes were determined to negatively influence the net release of dopamine at the brain reward site. Thousands of association studies have provided conclusive evidence of RDS-specific risks. Unfortunately, our laboratory is the only group investigating this potentially important DNA-directed test that could be used early in life to identify specific polymorphic risk alleles. With that in mind, several studies and reviews have been published. A sampling of these peer-reviewed articles provides the fundamental rationale to enable the futuristic applications of the GARS in all RDS behaviors to help identify risk [60,61,192,193,194,195,196,197,198,199,200,201,202,203,204,205,206,207,208,209,210,211,212].

In our opinion, subsequent large-scale genomics studies have had limited success in identifying alleles implicated in addiction and RDS. Although GWAS and next-generation sequencing are valuable genetic tools, the primary reason for this known limited success is resolvable. For example, GWAS identifies novel clusters of genes that may relate to an etiological root as a genetic antecedent to RDS behaviors such as AUD. However, we believe the next critical step following GWAS clusters is the subsequent convergence to individual candidate genes, despite their small contributions to the overall variance [213]. 

**Figure 4:**
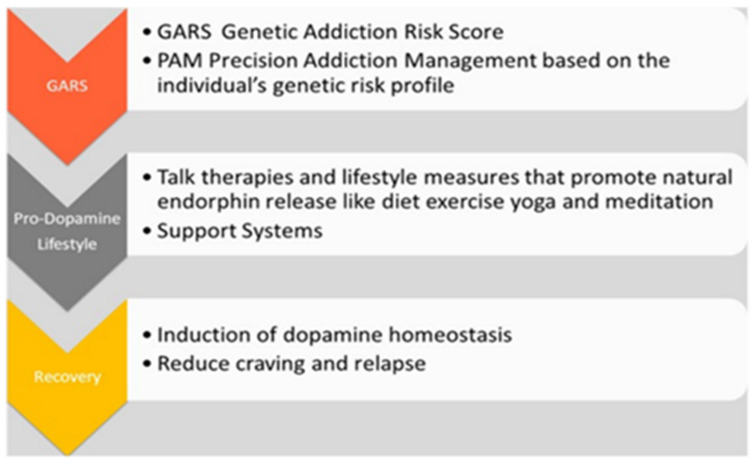
Precision addiction management platform [210] (with permission).

This precision addiction management technology (Figure 4) was developed to accurately identify genetic addiction risk severity using the GARS test and was awarded the first USA and foreign patents. As previously mentioned, Blum, Noble, and associates [60,187,189] published the first confirmed association of the DRD2 gene A1 allele with severe alcoholism and other RDS behaviors. Following this work, Blum et al. developed the GARS test and the prodopamine regulator, a precision DNA-guided nutraceutical neuronutrient (research ID code: KB220) (Figure 4).

## 3. Conclusions

**Figure 5:**
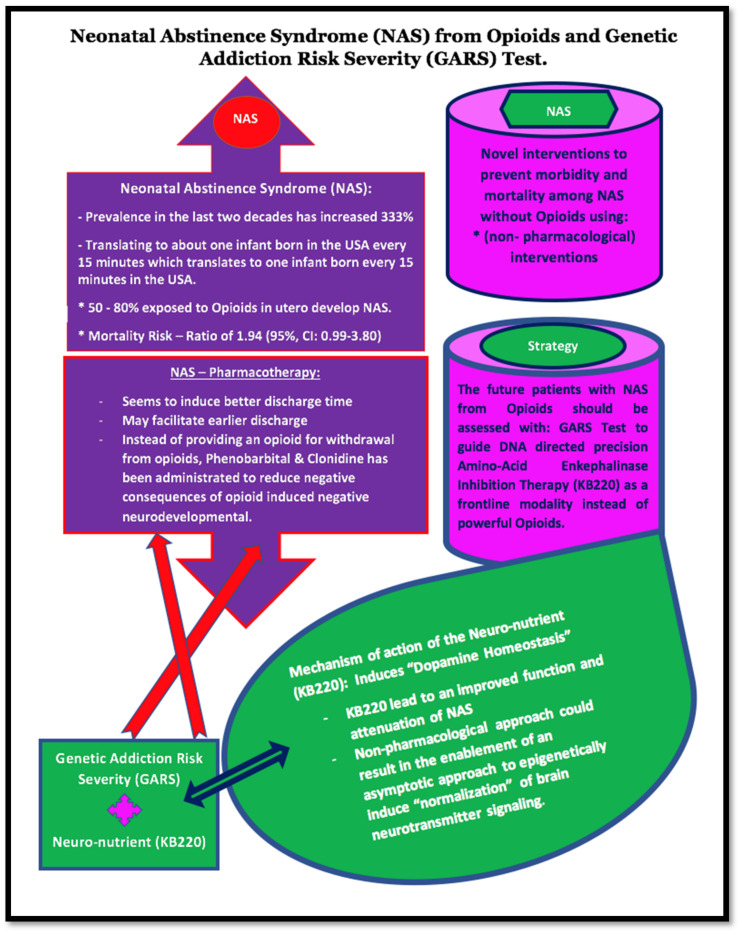
This schematic represents the paradigm shift required to circumvent the use of powerful opioids to treat opioid-induced NAS. We propose to couple the GARS test with DNA-directed precision KB220 therapy to detoxify and maintain patients with NAS using a non-opioid nutraceutical neuronutrient alternative.

Based on this extensive literature and additional research, we suggest that in the future, in addition to the current standard-of-care nonpharmacological therapies, patients with opioid-induced NAS should be assessed with the GARS test to guide DNA-directed precision amino-acid enkephalinase inhibition (KB220) therapy as a frontline modality instead of potent opioids (Figure 5).

## Figures and Tables

**Table 1:** Neuroadaptagen amino-acid therapy (NAAT) *.

GARS Listed Nutrient	Neuroadaptagen Target
D-Phenylalanine	Opioid peptides
L-Phenylalanine	Dopamine
L-tryptophan	Serotonin
L-Tyrosine	Dopamine
L-Glutamine (low dose)	GABA
Chromium	Serotonin
Rhodiola Rosea	COMT/MOA
Passionflower (low dose)	Benzodiazepine = GABA complex
Pyridoxine	Enzyme Catalyst

* Table 1 describes the ingredients and proposed neurotransmitter targets of KB220.

## Data Availability

Not applicable.

## References

[1] Kocherlakota P. (2014). Neonatal Abstinence Syndrome. Pediatrics.

[2] Isaacs K.R., Atreyapurapu S., Alyusuf A.H., Ledgerwood D.M., Finnegan L.P., Chang K.H.K., Ma T.X., Washio Y. (2021). Neonatal Outcomes after Combined Opioid and Nicotine Exposure in Utero: A Scoping Review. Int. J. Environ. Res. Public Health.

[3] Velez M., Jansson L.M. (2008). The Opioid Dependent Mother and Newborn Dyad: Nonpharmacologic Care. J. Addict. Med..

[4] Kandall S.R., Gartner L.M. (1974). Late presentation of drug withdrawal symptoms in newborns. Am. J. Dis. Child..

[5] Zankl A., Martin J., Davey J.G., Osborn D.A. (2021). Opioid treatment for opioid withdrawal in newborn infants. Cochrane Database Syst. Rev..

[6] (2021). NSDUH Annual National Report. https://www.samhsa.gov/data/report/2020-nsduh-annual-national-report.

[7] (2022). Death Rate Maps & Graphs. https://www.cdc.gov/drugoverdose/deaths/index.html.

[8] U.S (2022). Overdose Deaths in 2021 Increased Half as Much as in 2020—But Are Still Up 15%. https://www.cdc.gov/nchs/pressroom/nchs_press_releases/2022/202205.htm.

[9] Hirai A.H., Ko J.Y., Owens P.L., Stocks C., Patrick S.W. (2021). Neonatal Abstinence Syndrome and Maternal Opioid-Related Diagnoses in the US, 2010–2017. JAMA.

[10] Patrick S.W., Faherty L.J., Dick A.W., Scott T.A., Dudley J., Stein B.D. (2019). Association Among County-Level Economic Factors, Clinician Supply, Metropolitan or Rural Location, and Neonatal Abstinence Syndrome. JAMA.

[11] Siu A., Robinson C.A. (2014). Neonatal Abstinence Syndrome: Essentials for the Practitioner. J. Pediatr. Pharmacol. Ther..

[12] Patrick S.W., Barfield W.D., Poindexter B.B., Committee on Fetus and Newborn, Committee on Substance Use and Prevention (2020). Neonatal Opioid Withdrawal Syndrome. Pediatrics.

[13] Howard M.B., Schiff D.M., Penwill N., Si W., Rai A., Wolfgang T., Moses J.M., Wachman E.M. (2017). Impact of Parental Presence at Infants’ Bedside on Neonatal Abstinence Syndrome. Hosp. Pediatr..

[14] Macmillan K.D.L., Rendon C.P., Verma K., Riblet N., Washer D.B., Holmes A.V. (2018). Association of Rooming-in with Outcomes for Neonatal Abstinence Syndrome. JAMA Pediatr..

[15] Abrahams R.R., Kelly S.A., Payne S., Thiessen P.N., Mackintosh J., Janssen P.A. (2007). Rooming-in compared with standard care for newborns of mothers using methadone or heroin. Can. Fam. Physician.

[16] Jansson L.M., Academy of Breastfeeding Medicine Protocol Committee (2009). ABM Clinical Protocol #21: Guidelines for Breastfeeding and the Drug-Dependent Woman. Breastfeed. Med..

[17] Sachs H.C., Frattarelli D.A.C., Galinkin J.L., Green T.P., Johnson T., Neville K., Paul I.M., Anker J.V.D., Committee on Drugs (2013). The Transfer of Drugs and Therapeutics Into Human Breast Milk: An Update on Selected Topics. Pediatrics.

[18] Favara M.T., Carola D., Jensen E., Cook A., Genen L., Dysart K., Greenspan J.S., Aghai Z.H. (2019). Maternal breast milk feeding and length of treatment in infants with neonatal abstinence syndrome. J. Perinatol..

[19] Jansson L.M., Choo R., Velez M.L., Harrow C., Schroeder J.R., Shakleya D.M., Huestis M.A. (2008). Methadone Maintenance and Breastfeeding in the Neonatal Period. Pediatrics.

[20] Abdel-Latif M.E., Pinner J., Clews S., Cooke F., Lui K., Oei J. (2006). Effects of Breast Milk on the Severity and Outcome of Neonatal Abstinence Syndrome Among Infants of Drug-Dependent Mothers. Pediatrics.

[21] Isemann B., Meinzen-Derr J., Akinbi H. (2011). Maternal and neonatal factors impacting response to methadone therapy in infants treated for neonatal abstinence syndrome. J. Perinatol..

[22] Welle-Strand G.K., Skurtveit S., Jansson L.M., Bakstad B., Bjarkø L., Ravndal E. (2013). Breastfeeding reduces the need for withdrawal treatment in opioid-exposed infants. Acta Paediatr..

[23] Ilett K.F., Hackett L.P., Gower S., Doherty D.A., Hamilton D., Bartu A.E. (2012). Estimated Dose Exposure of the Neonate to Buprenorphine and Its Metabolite Norbuprenorphine via Breastmilk During Maternal Buprenorphine Substitution Treatment. Breastfeed. Med..

[24] Jansson L.M., Spencer N., McConnell K., Velez M., Tuten M., Harrow C.A., Jones H.E., Swortwood M.J., Barnes A.J., Scheidweiler K.B. (2016). Maternal Buprenorphine Maintenance and Lactation. J. Hum. Lact..

[25] Kaltenbach K., O’Grady K.E., Heil S.H., Salisbury A.L., Coyle M.G., Fischer G., Martin P.R., Stine S., Jones H.E. (2018). Prenatal exposure to methadone or buprenorphine: Early childhood developmental outcomes. Drug Alcohol Depend..

[26] Hall E.S., Wexelblatt S.L., Crowley M., Grow J.L., Jasin L.R., Klebanoff M.A., McClead R.E., Meinzen-Derr J., Mohan V.K., Stein H. (2014). A Multicenter Cohort Study of Treatments and Hospital Outcomes in Neonatal Abstinence Syndrome. Pediatrics.

[27] Zimmermann U., Rudin C., Duò A., Held L., Bucher H.U., On behalf of the Swiss neonatal abstinence syndrome study group (2019). Treatment of opioid withdrawal in neonates with morphine, phenobarbital, or chlorpromazine: A randomized double-blind trial. Eur. J. Pediatr..

[28] Osborn D.A., Jeffery H.E., Cole M.J. (2010). Opiate treatment for opiate withdrawal in newborn infants. Cochrane Database Syst. Rev..

[29] Davis J.M., Shenberger J., Terrin N., Breeze J.L., Hudak M., Wachman E.M., Marro P., Oliveira E.L., Harvey-Wilkes K., Czynski A. (2018). Comparison of Safety and Efficacy of Methadone vs Morphine for Treatment of Neonatal Abstinence Syndrome: A Randomized Clinical Trial. JAMA Pediatr..

[30] Tolia V.N., Murthy K., Bennett M.M., Greenberg R.G., Benjamin D.K., Smith P.B., Clark R.H. (2018). Morphine vs Methadone Treatment for Infants with Neonatal Abstinence Syndrome. J. Pediatr..

[31] Kraft W.K. (2017). Buprenorphine in Neonatal Abstinence Syndrome. Clin. Pharmacol. Ther..

[32] Kraft W.K., Adeniyi-Jones S.C., Chervoneva I., Greenspan J.S., Abatemarco D., Kaltenbach K., Ehrlich M.E. (2017). Buprenorphine for the Treatment of the Neonatal Abstinence Syndrome. N. Engl. J. Med..

[33] Disher T., Gullickson C., Singh B., Cameron C., Boulos L., Beaubien L., Campbell-Yeo M. (2019). Pharmacological Treatments for Neonatal Abstinence Syndrome. JAMA Pediatr..

[34] Cleary B.J., Donnelly J., Strawbridge J., Gallagher P.J., Fahey T., Clarke M., Murphy D.J. (2010). Methadone dose and neonatal abstinence syndrome-systematic review and meta-analysis. Addiction.

[35] Bakstad B., Sarfi M., Welle-Strand G.K., Ravndal E. (2009). Opioid Maintenance Treatment during Pregnancy: Occurrence and Severity of Neonatal Abstinence Syndrome: A national prospective study. Eur. Addict. Res..

[36] Streetz V.N., Gildon B.L., Thompson D.F. (2016). Role of Clonidine in Neonatal Abstinence Syndrome. Ann. Pharmacother..

[37] Merhar S.L., Ounpraseuth S., Devlin L.A., Poindexter B.B., Young L.W., Berkey S.D., Crowley M., Czynski A.J., Kiefer A.S., Whalen B.L. (2021). Phenobarbital and Clonidine as Secondary Medications for Neonatal Opioid Withdrawal Syndrome. Pediatrics.

[38] Bio L.L., Siu A., Poon C.Y. (2011). Update on the pharmacologic management of neonatal abstinence syndrome. J. Perinatol..

[39] Forcelli P., Janssen M.J., Stamps L.A., Sweeney C., Vicini S., Gale K. (2010). Therapeutic strategies to avoid long-term adverse outcomes of neonatal antiepileptic drug exposure. Epilepsia.

[40] Forcelli P.A., Kim J., Kondratyev A., Gale K. (2011). Pattern of antiepileptic drug-induced cell death in limbic regions of the neonatal rat brain. Epilepsia.

[41] Forcelli P.A., Kozlowski R., Snyder C., Kondratyev A., Gale K. (2012). Effects of Neonatal Antiepileptic Drug Exposure on Cognitive, Emotional, and Motor Function in Adult Rats. J. Pharmacol. Exp. Ther..

[42] Piccotti L., Voigtman B., Vongsa R., Nellhaus E.M., Rodriguez K.J., Davies T.H., Quirk S. (2019). Neonatal Opioid Withdrawal Syndrome: A Developmental Care Approach. Neonatal Netw..

[43] Agthe A.G., Kim G.R., Mathias K.B., Hendrix C.W., Chavez-Valdez R., Jansson L., Lewis T.R., Yaster M., Gauda E.B. (2009). Clonidine as an Adjunct Therapy to Opioids for Neonatal Abstinence Syndrome: A Randomized, Controlled Trial. Pediatrics.

[44] Johnson K., Gerada C., Greenough A. (2003). Treatment of neonatal abstinence syndrome. Arch. Dis. Child. Fetal Neonatal Ed..

[45] Blum K., Modestino E.J., Gondre-Lewis M.C., Baron D., Thanos P.K., Downs B.W., Siwicki D., Lott L., Braverman E.R., Moran M. (2018). Dopamine Regulator (KB220) A Fifty Year Sojourn to Combat Reward Deficiency Syndrome (RDS): Evidence Based Bibliography (Annotated). CPQ Neurol. Psychol..

[46] Czynski A.J., Davis J.M., Dansereau L.M., Engelhardt B., Marro P., Bogen D.L., Hudak M.L., Shenberger J., Wachman E.M., Oliveira E.L. (2020). Neurodevelopmental Outcomes of Neonates Randomized to Morphine or Methadone for Treatment of Neonatal Abstinence Syndrome. J. Pediatr..

[47] Haight S.C., Ko J.Y., Tong V.T., Bohm M.K., Callaghan W.M. (2018). Opioid use disorder documented at delivery hospitalization—United States, 1999–2014. Morb. Mortal. Wkly. Rep..

[48] Boardman J.P., Mactier H., Devlin L.A. (2021). Opioids and the developing brain: Time to rethink perinatal care for infants of opioid-dependent mothers. Arch. Dis. Child. Fetal Neonatal Ed..

[49] Hoogen N.J.V.D., Kwok C.H.T., Trang T. (2021). Identifying the Neurodevelopmental Differences of Opioid Withdrawal. Cell Mol. Neurobiol..

[50] Witt C.E., Rudd K., Bhatraju P., Rivara F.P., Hawes S.E., Weiss N.S. (2017). Neonatal abstinence syndrome and early childhood morbidity and mortality in Washington state: A retrospective cohort study. J. Perinatol..

[51] Lu H., Rosenbaum S. (2014). Developmental Pharmacokinetics in Pediatric Populations. J. Pediatr. Pharmacol. Ther..

[52] Kerui G., Jasmin L. (2018). Dual effects of brain sparing opioid in newborn rats: Analgesia and hyperalgesia. Neurobiol. Pain.

[53] Jackson H.J., Lopez C., Miller S., Engelhardt B. (2019). A Scoping Review of Acupuncture as a Potential Intervention for Neonatal Abstinence Syndrome. Med. Acupunct..

[54] Filippelli A.C., White L.F., Spellman L.W., Broderick M., Highfield E.S., Sommers E., Gardiner P. (2012). Non-insertive Acupuncture and Neonatal Abstinence Syndrome: A Case Series from an Inner-city Safety Net Hospital. Glob. Adv. Health Med..

[55] Mangat A., Schmölzer G., Kraft W. (2019). Pharmacological and non-pharmacological treatments for the Neonatal Abstinence Syndrome (NAS). Semin. Fetal Neonatal Med..

[56] Brocato B., Lewis D., Eyal F., Baker S., Armistead C., Kaye A.D., Cornett E.M., Whitehurst R.M. (2022). The Impact of a Prenatal Education Program for Opioid-Dependent Mothers on Breastfeeding Rates of Infants at Risk for Neonatal Abstinence Syndrome. Adv. Ther..

[57] Blum K., Whitney D., Fried L., Febo M., Waite R.L., Braverman E.R., Dushaj K., Li M., Giordano J., Demetrovics Z. (2016). Hypothesizing that a Pro-Dopaminergic Regulator (KB220z™ Liquid Variant) can Induce “Dopamine Homeostasis” and Provide Adjunctive Detoxification Benefits in Opiate/Opioid Dependence. Clin. Med. Rev. Case Rep..

[58] Blum K., Trachtenberg M.C., Ramsay J.C. (1988). Improvement of Inpatient Treatment of the Alcoholic as a Function of Neurotransmitter Restoration: A Pilot Study. Int. J. Addict..

[59] Poisson C.L., Engel L., Saunders B.T. (2021). Dopamine Circuit Mechanisms of Addiction-Like Behaviors. Front. Neural Circuits.

[60] Blum K., Noble E.P., Sheridan P.J., Montgomery A., Ritchie T., Jagadeeswaran P., Nogami H., Briggs A.H., Cohn J.B. (1990). Allelic association of human dopamine D2 receptor gene in alcoholism. JAMA.

[61] Blum K., Kazmi S., Modestino E., Downs B., Bagchi D., Baron D., McLaughlin T., Green R., Jalali R., Thanos P. (2021). A Novel Precision Approach to Overcome the “Addiction Pandemic” by Incorporating Genetic Addiction Risk Severity (GARS) and Dopamine Homeostasis Restoration. J. Pers. Med..

[62] Swanson L. (1982). The projections of the ventral tegmental area and adjacent regions: A combined fluorescent retrograde tracer and immunofluorescence study in the rat. Brain Res. Bull..

[63] Ikemoto S. (2007). Dopamine reward circuitry: Two projection systems from the ventral midbrain to the nucleus accum-bens-olfactory tubercle complex. Brain Res. Rev..

[64] Olson V.G., Nestler E.J. (2007). Topographical organization of GABAergic neurons within the ventral tegmental area of the rat. Synapse.

[65] Nair-Roberts R., Chatelain-Badie S., Benson E., White-Cooper H., Bolam J., Ungless M. (2008). Stereological estimates of dopaminergic, GABAergic and glutamatergic neurons in the ventral tegmental area, substantia nigra and retrorubral field in the rat. Neuroscience.

[66] Bouarab C., Thompson B., Polter A.M. (2019). VTA GABA Neurons at the Interface of Stress and Reward. Front. Neural Circuits.

[67] Beckstead R.M., Domesick V.B., Nauta W.J. (1979). Efferent connections of the substantia nigra and ventral tegmental area in the rat. Brain Res..

[68] Fields H.L., Hjelmstad G.O., Margolis E.B., Nicola S.M. (2007). Ventral Tegmental Area Neurons in Learned Appetitive Behavior and Positive Reinforcement. Annu. Rev. Neurosci..

[69] Britt J.P., Benaliouad F., McDevitt R.A., Stuber G.D., Wise R.A., Bonci A. (2012). Synaptic and Behavioral Profile of Multiple Glutamatergic Inputs to the Nucleus Accumbens. Neuron.

[70] Gerfen C.R. (2003). D1 Dopamine Receptor Supersensitivity in the Dopamine-Depleted Striatum Animal Model of Parkinson’s Disease. Neuroscientist.

[71] Kupchik Y.M., Brown R., Heinsbroek J., Lobo M.K., Schwartz D.J., Kalivas P.W. (2015). Coding the direct/indirect pathways by D1 and D2 receptors is not valid for accumbens projections. Nat. Neurosci..

[72] Collins A.L., Saunders B.T. (2020). Heterogeneity in striatal dopamine circuits: Form and function in dynamic reward seeking. J. Neurosci. Res..

[73] Koob G.F., Bloom F.E. (1988). Cellular and Molecular Mechanisms of Drug Dependence. Science.

[74] Ahmed S.H., Koob G.F. (1998). Transition from Moderate to Excessive Drug Intake: Change in Hedonic Set Point. Science.

[75] Lobo M.K., Covington H.E., Chaudhury D., Friedman A.K., Sun H., Damez-Werno D., Dietz D.M., Zaman S., Koo J.W., Kennedy P.J. (2010). Cell Type–Specific Loss of BDNF Signaling Mimics Optogenetic Control of Cocaine Reward. Science.

[76] Thompson D., Martini L., Whistler J.L. (2010). Altered Ratio of D1 and D2 Dopamine Receptors in Mouse Striatum Is Associated with Behavioral Sensitization to Cocaine. PLoS ONE.

[77] Oliver R.J., Purohit D.C., Kharidia K.M., Mandyam C.D. (2019). Transient Chemogenetic Inhibition of D1-MSNs in the Dorsal Striatum Enhances Methamphetamine Self-Administration. Brain Sci..

[78] Björklund A., Dunnett S. (2007). Dopamine neuron systems in the brain: An update. Trends Neurosci..

[79] Lammel S., Lim B.K., Malenka R.C. (2014). Reward and aversion in a heterogeneous midbrain dopamine system. Neuropharmacology.

[80] Morales M., Margolis E.B. (2017). Ventral tegmental area: Cellular heterogeneity, connectivity and behaviour. Nat. Rev. Neurosci..

[81] Cox J., Witten I.B. (2019). Striatal circuits for reward learning and decision-making. Nat. Rev. Neurosci..

[82] Haber S.N., Fudge J.L., McFarland N.R. (2000). Striatonigrostriatal Pathways in Primates Form an Ascending Spiral from the Shell to the Dorsolateral Striatum. J. Neurosci..

[83] Hassani O.K., Cromwell H., Schultz W. (2001). Influence of Expectation of Different Rewards on Behavior-Related Neuronal Activity in the Striatum. J. Neurophysiol..

[84] Everitt B.J. (2014). Neural and psychological mechanisms underlying compulsive drug seeking habits and drug memories—Indications for novel treatments of addiction. Eur. J. Neurosci..

[85] Everitt B.J., Robbins T. (2005). Neural systems of reinforcement for drug addiction: From actions to habits to compulsion. Nat. Neurosci..

[86] Wise R.A. (2005). Forebrain substrates of reward and motivation. J. Comp. Neurol..

[87] Wise R.A. (2009). Roles for nigrostriatal—Not just mesocorticolimbic—Dopamine in reward and addiction. Trends Neurosci..

[88] Berridge K.C. (2007). The debate over dopamine’s role in reward: The case for incentive salience. Psychopharmacology.

[89] Flagel S.B., Clark J.J., Robinson T.E., Mayo L., Czuj A., Willuhn I., Akers C.A., Clinton S.M., Phillips P.E.M., Akil H. (2011). A selective role for dopamine in stimulus–reward learning. Nature.

[90] Tiffany S.T. (1990). A cognitive model of drug urges and drug-use behavior: Role of automatic and nonautomatic processes. Psychol. Rev..

[91] Willuhn I., Burgeno L.M., Everitt B.J., Phillips P.E.M. (2012). Hierarchical recruitment of phasic dopamine signaling in the striatum during the progression of cocaine use. Proc. Natl. Acad. Sci. USA.

[92] Willuhn I., Burgeno L., Groblewski P.A., Phillips P.E.M. (2014). Excessive cocaine use results from decreased phasic dopamine signaling in the striatum. Nat. Neurosci..

[93] Ostlund S.B. (2019). The push and pull of dopamine in cue-reward learning. Anim. Learn. Behav..

[94] Ostlund S.B., LeBlanc K.H., Kosheleff A.R., Wassum K.M., Maidment N.T. (2014). Phasic Mesolimbic Dopamine Signaling Encodes the Facilitation of Incentive Motivation Produced by Repeated Cocaine Exposure. Neuropsychopharmacology.

[95] Hogarth L. (2020). Addiction is driven by excessive goal-directed drug choice under negative affect: Translational critique of habit and compulsion theory. Neuropsychopharmacology.

[96] Volkow N., Fowler J., Wang G., Baler R., Telang F. (2009). Imaging dopamine’s role in drug abuse and addiction. Neuropharmacology.

[97] Volkow N.D., Wang G.-J., Telang F., Fowler J.S., Logan J., Childress A.-R., Jayne M., Ma Y., Wong C. (2006). Cocaine Cues and Dopamine in Dorsal Striatum: Mechanism of Craving in Cocaine Addiction. J. Neurosci..

[98] Volkow N.D., Wise R.A., Baler R. (2017). The dopamine motive system: Implications for drug and food addiction. Nat. Rev. Neurosci..

[99] Wang Y.-C., Ho U.-C., Ko M.-C., Liao C.-C., Lee L.-J. (2012). Differential neuronal changes in medial prefrontal cortex, basolateral amygdala and nucleus accumbens after postweaning social isolation. Anat. Embryol..

[100] Vandaele Y., Ahmed S.H. (2021). Habit, choice, and addiction. Neuropsychopharmacology.

[101] Aarts E., van Holstein M., Cools R. (2011). Striatal Dopamine and the Interface between Motivation and Cognition. Front. Psychol..

[102] Bardo M.T., Neisewander J., Kelly T.H. (2013). Individual Differences and Social Influences on the Neurobehavioral Pharmacology of Abused Drugs. Pharmacol. Rev..

[103] Bello E.P., Mateo Y., Gelman D.M., Noaín D., Shin J.H., Low M.J., Alvarez V., Lovinger D.M., Rubinstein M. (2011). Cocaine supersensitivity and enhanced motivation for reward in mice lacking dopamine D2 autoreceptors. Nat. Neurosci..

[104] Blum K., Braverman E.R., Holder J.M., Lubar J.F., Monastra V.J., Miller D., Lubar J.O., Chen T.J., Comings D.E. (2000). The Reward Deficiency Syndrome: A Biogenetic Model for the Diagnosis and Treatment of Impulsive, Addictive and Compulsive Behaviors. J. Psychoact. Drugs.

[105] Blum K., Thanos P.K., Oscar-Berman M., Febo M., Baron D., Badgaiyan R.D., Gardner E., Demetrovics Z., Fahlke C., Haberstick B.C. (2015). Dopamine in the Brain: Hypothesizing Surfeit or Deficit Links to Reward and Addiction. J. Reward Defic. Syndr..

[106] Buckholtz J.W., Treadway M.T., Cowan R.L., Woodward N.D., Li R., Ansari M.S., Baldwin R.M., Schwartzman A.N., Shelby E.S., Smith C.E. (2010). Dopaminergic Network Differences in Human Impulsivity. Science.

[107] Burton A.C., Nakamura K., Roesch M.R. (2015). From ventral-medial to dorsal-lateral striatum: Neural correlates of reward-guided decision-making. Neurobiol. Learn. Mem..

[108] Di Chiara G., Imperato A. (1988). Drugs abused by humans preferentially increase synaptic dopamine concentrations in the mesolimbic system of freely moving rats. Proc. Natl. Acad. Sci. USA.

[109] Griffiths R.R., Brady J.V., Bradford L.D. (1979). Predicting the Abuse Liability of Drugs with Animal Drug Self-Administration Procedures: Psychomotor Stimulants and Hallucinogens. Adv. Behav. Pharmacol..

[110] Ito R., Dalley J., Howes S.R., Robbins T., Everitt B. (2000). Dissociation in Conditioned Dopamine Release in the Nucleus Accumbens Core and Shell in Response to Cocaine Cues and during Cocaine-Seeking Behavior in Rats. J. Neurosci..

[111] Ito R., Dalley J., Robbins T., Everitt B. (2002). Dopamine Release in the Dorsal Striatum during Cocaine-Seeking Behavior under the Control of a Drug-Associated Cue. J. Neurosci..

[112] Leyton M. (2017). Altered dopamine transmission as a familial risk trait for addictions. Curr. Opin. Behav. Sci..

[113] Leyton M., Vezina P. (2014). Dopamine ups and downs in vulnerability to addictions: A neurodevelopmental model. Trends Pharmacol. Sci..

[114] Murray J.E., Belin D., Everitt B. (2012). Double Dissociation of the Dorsomedial and Dorsolateral Striatal Control Over the Acquisition and Performance of Cocaine Seeking. Neuropsychopharmacology.

[115] Nutt D.J., Lingford-Hughes A., Erritzoe D., Stokes P. (2015). The dopamine theory of addiction: 40 years of highs and lows. Nat. Rev. Neurosci..

[116] Ray L.A., Courtney K.E., Hutchison K.E., MacKillop J., Galvan A., Ghahremani D.G. (2014). Initial evidence that OPRM1 genotype moderates ventral and dorsal striatum functional connectivity during alcohol cues. Alcohol. Clin. Exp. Res..

[117] Ritz M.C., Lamb R.J., Goldberg S.R., Kuhar M.J. (1987). Cocaine receptors on dopamine transporters are related to self-administration of cocaine. Science.

[118] Venniro M., Caprioli D., Shaham Y. (2016). Animal models of drug relapse and craving: From drug priming-induced rein-statement to incubation of craving after voluntary abstinence. Prog. Brain Res..

[119] Ztaou S., Lhost J., Watabe I., Torromino G., Amalric M. (2018). Striatal cholinergic interneurons regulate cognitive and affective dysfunction in partially dopamine-depleted mice. Eur. J. Neurosci..

[120] Wachman E.M., Hayes M.J., Brown M.S., Paul J., Harvey-Wilkes K., Terrin N., Huggins G.S., Aranda J.V., Davis J.M. (2013). Association of OPRM1 and COMT Single-Nucleotide Polymorphisms with Hospital Length of Stay and Treatment of Neonatal Abstinence Syndrome. JAMA.

[121] Mague S.D., Blendy J.A. (2010). OPRM1 SNP (A118G): Involvement in disease development, treatment response, and animal models. Drug Alcohol Depend..

[122] Wachman E.M., Hayes M.J., Lester B.M., Terrin N., Brown M.S., Nielsen D., Davis J.M. (2014). Epigenetic Variation in the Mu-Opioid Receptor Gene in Infants with Neonatal Abstinence Syndrome. J. Pediatr..

[123] Blum K., Berman M.O., Jacobs W., McLaughlin T., Gold M.S. (2014). Buprenorphine Response as a Function of Neurogenetic Polymorphic Antecedents: Can Dopamine Genes Affect Clinical Outcomes in Reward Deficiency Syndrome (RDS)?. J. Addict. Res. Ther..

[124] Kurath-Koller S., Pansy J., Mileder L.P., Schmölzer G.M., Urlesberger B., Raith W. (2016). Active Somatic and Psychic Ear Acupuncture Points in Newborn Infants with Neonatal Abstinence Syndrome. J. Altern. Complement. Med..

[125] Doughterty P., Dafny N. (1989). Trans-cranial electrical stimulation attenuates the severity of naloxone-precipitated morphine withdrawal in rats. Life Sci..

[126] Gold M.S. (1993). Opiate Addiction and the Locus Coeruleus: The Clinical Utility of Clonidine, Naltrexone, Methadone, and Buprenorphine. Psychiatr. Clin. N. Am..

[127] Tucker K.R., Cavolo S.L., Levitan E.S. (2016). Elevated mitochondria-coupled NAD(P)H in endoplasmic reticulum of dopamine neurons. Mol. Biol. Cell.

[128] Hodebourg R., Murray J.E., Fouyssac M., Puaud M., Everitt B.J., Belin D. (2019). Heroin seeking becomes dependent on dorsal striatal dopaminergic mechanisms and can be decreased by N-acetylcysteine. Eur. J. Neurosci..

[129] Van Draanen J., Tsang C., Mitra S., Phuong V., Murakami A., Karamouzian M., Richardson L. (2021). Mental disorder and opioid overdose: A systematic review. Soc. Psychiatry.

[130] Marshall B.D., Green T.C., Yedinak J.L., Hadland S.E. (2016). Harm reduction for young people who use prescription opioids extra-medically: Obstacles and opportunities. Int. J. Drug Policy.

[131] Blum K., Baron D. (2019). Opioid Substitution Therapy: Achieving Harm Reduction While Searching for a Prophylactic Solution. Curr. Pharm. Biotechnol..

[132] Blum K., Bowirrat A., Braverman E.R., Baron D., Cadet J.L., Kazmi S., Elman I., Thanos P.K., Badgaiyan R.D., Downs W.B. (2021). Reward Deficiency Syndrome (RDS): A Cytoarchitectural Common Neurobiological Trait of All Addictions. Int. J. Environ. Res. Public Health.

[133] Blum K., Badgaiyan R.D. (2021). Translational and Molecular Cytoarchitectural Genetic Guided Therapy to Induce Dopamine Ho-meostatic Neuro-signaling in Reward Deficiency and Associated Drug and Behavioral Addiction Seeking: A 60 Year Sojourn the Future is Now. EC Psychol. Psychiatr..

[134] Febo M., Blum K., Badgaiyan R.D., Perez P.D., Colon-Perez L.M., Thanos P.K., Ferris C.F., Kulkarni P., Giordano J., Baron D. (2017). Enhanced functional connectivity and volume between cognitive and reward centers of naïve rodent brain produced by pro-dopaminergic agent KB220Z. PLoS ONE.

[135] Blum K., Elston S.F., DeLallo L., Briggs A.H., Wallace J.E. (1983). Ethanol acceptance as a function of genotype amounts of brain [Met]enkephalin. Proc. Natl. Acad. Sci. USA.

[136] Blum K., Briggs A.H., Trachtenberg M.C., Delallo L., Wallace J.E. (1987). Enkephalinase inhibition: Regulation of ethanol intake in genetically predisposed mice. Alcohol.

[137] Brown R.J., Blum K., Trachtenberg M.C. (1990). Neurodynamics of Relapse Prevention: A Neuronutrient Approach to Outpatient DUI Offenders. J. Psychoact. Drugs.

[138] Blum K., Trachtenberg M.C., Elliott C.E., Dingler M.L., Sexton R.L., Samuels A.I., Cataldie L. (1988). Enkephalinase inhibition and precursor amino acid loading improves inpatient treatment of alcohol and polydrug abusers: Double-blind placebo-controlled study of the nutri-tional adjunct SAAVE. Alcohol.

[139] Blum K., Allison D., Trachtenberg M.C., Williams R.W. (1988). Reduction of both drug hunger and withdrawal against advice rate of cocaine abusers in a 30-day inpatient treatment program by the neuronutrient Tropamine. Curr. Ther. Res..

[140] Blum K., Trachtenberg M.C., Cook D.W. (1990). Neuronutrient effects on weight loss in carbohydrate bingers; an open clinical trial. Curr. Ther. Res..

[141] Cold J.A. (1996). NeuRecover-SATM in the Treatment of Cocaine Withdrawal and Craving: A Pilot Study Clin. Drug Investig..

[142] DeFrance J.F., Hymel C., Trachtenberg M.C., Ginsberg L.D., Schweitzer F.C., Estes S., Chen T.J., Braverman E.R., Cull J.G., Blum K. (1997). Enhancement of attention pro-cessing by Kantroll in healthy humans: A pilot study. Clin. Electroencephalogr..

[143] Chen T.J.H., Blum K., Payte J.T., Schoolfield J., Hopper D., Stanford M., Braverman E.R. (2004). Narcotic antagonists in drug dependence: Pilot study showing enhancement of compliance with SYN-10, amino-acid precursors and enkephalinase inhibition therapy. Med. Hypotheses.

[144] Blum K., Chen T.J., Meshkin B., Downs B.W., Gordon C.A., Blum S., Mengucci J.F., Braverman E.R., Arcuri V., Varshavskiy M. (2006). Reward deficiency syndrome in obesity: A pre-liminary cross-sectional trial with a Genotrim variant. Adv. Ther..

[145] Chen T.J.H., Blum K., Waite R.L., Meshkin B., Schoolfield J., Downs B.W., Braverman E.E., Arcuri V., Varshavskiy M., Blum S.H. (2007). Gene\Narcotic Attenuation Program attenuates substance use disorder, a clinical subtype of reward deficiency syndrome. Adv. Ther..

[146] Blum K., Chen T.J.H., Downs B.W., Meshkin B., Blum S.H., Martinez Pons M. (2007). Synaptamine (SG8839), TM an Ami-no-Acid Enkephalinase Inhibition Nutraceutical Improves Recovery of Alcoholics, A Subtype of Reward Deficiency Syndrome (RDS). Trends Appl. Sci. Res..

[147] Chen T.J., Blum K., Kaats G., Braverman E.R., Eisenberg A., Sherman M., Davis K., Comings D.E., Wood R., Pullin D. (2007). Chromium Picolinate (Crp) A putative An-ti-Obesity Nutrient Induces Changes in Body Composition as Function Of The Taq1 Dopamine D2 Receptor Gene. Gene Ther. Mol. Biol..

[148] Blum K., Chen T.J., Williams L., Chen A.L., Downs W.B., Waite R.L., Huntington T., Sims S., Prihoda R., Reinking J. (2008). A short term pilot open label study to evaluate efficacy and safety of LG839, a customized DNA directed nutraceutical in obesity: Exploring Nutrigenomics. Gene Ther. Mol. Biol..

[149] Blum K., Chen A.L.C., Chen T.J.H., Rhoades P., Prihoda T.J., Downs B.W., Waite R.L., Williams L., Braverman E.R., Braverman D. (2008). LG839: Anti-obesity effects and polymorphic gene correlates of reward deficiency syndrome. Adv. Ther..

[150] Blum K., Chen T.J., Chen A.L., Rhoades P., Prihoda T.J., Downs B.W., Bagchi D., Bagchi M., Blum S.H., Williams L. (2008). Dopamine D2 Receptor Taq A1 allele predicts treatment compliance of LG839 in a subset analysis of pilot study in the Netherlands. Gene Ther. Mol. Biol..

[151] Blum K., Chen A.L., Chen T.J., Bowirrat A., Waite R.L., Kerner M., Blum S.H., Downs B.W., Savarimuthu S., Rhoades P. (2009). Putative targeting of Dopamine D2 receptor function in Reward Deficiency Syndrome (RDS) by Synaptamine Complex™ Variant (KB220): Clinical trial showing anti anxiety effects. Gene Ther. Mol. Biol..

[152] Braverman E.R., Braverman D., Acrui V., Kerner M., Downs B.W., Blum K. (2010). Targeting noradrenergic and dopaminergic mechanistic sites, hormonal deficiency repletion therapy and exercise: A case report. Am. J. Bariatr. Med..

[153] Miller D.K., Bowirrat A., Manka M., Miller M., Stokes S., Manka D., Allen C., Gant C., Downs B.W., Smolen A. (2010). Acute intravenous synaptamine complex variant KB220™ “normalizes” neurological dysregulation in patients during protracted abstinence from alcohol and opiates as ob-served using quantitative electroencephalographic and genetic analysis for reward polymorphisms: Part 1, pilot study with 2 case reports. Postgrad Med..

[154] Blum K., Chen T.J.H., Morse S., Giordano J., Chen A.L.C., Thompson J., Allen C., Smolen A., Lubar J., Stice E. (2010). Overcoming qEEG Abnormalities and Reward Gene Deficits during Protracted Abstinence in Male Psychostimulant and Polydrug Abusers Utilizing Putative Dopamine D_2_Agonist Therapy: Part 2. Postgrad. Med..

[155] Chen D., Liu Y., He W., Wang H., Wang Z. (2012). Neurotransmitter-precursor-supplement intervention for detoxified heroin addicts. J. Huazhong Univ. Sci. Technol..

[156] Miller M., Chen A.L., Stokes S.D., Silverman S., Bowirrat A., Manka M., Manka D., Miller D.K., Perrine K., Chen T.J. (2012). Early Intervention of Intravenous KB220IV- Neuroadaptagen Amino-Acid Therapy (NAAT)™ Improves Behavioral Outcomes in a Residential Addiction Treatment Program: A Pilot Study. J. Psychoact. Drugs.

[157] Blum K., Oscar-Berman M., Femino J., Waite R.L., Benya L., Giordano J., Borsten J., Downs W.B., Braverman E.R., Loehmann R. (2013). Withdrawal from Buprenorphine/Naloxone and Maintenance with a Natural Dopaminergic Agonist: A Cautionary Note. J. Addict. Res. Ther..

[158] McLaughlin T., Blum K., Berman M.O., Febo M., Agan G., Fratantonio J.L., Simpatico T., Gold M.S. (2015). Putative dopamine agonist (KB220Z) attenuates lucid nightmares in PTSD patients: Role of enhanced brain reward functional connectivity and homeostasis redeeming joy. J. Behav. Addict..

[159] McLaughlin T., Blum K., Oscar-Berman M., Febo M., Demetrovics Z., Agan G., Fratantonio J., Gold M.S. (2015). Using the Neuroadaptagen KB200z™ to Ameliorate Terrifying, Lucid Nightmares in RDS Patients: The Role of Enhanced, Brain-Reward, Functional Connectivity and Dopaminergic Homeostasis. J. Reward Defic. Syndr..

[160] McLaughlin T., Febo M., Badgaiyan R.D., Barh D., Dushaj K., Braverman E.R., Li M., Madigan M.A., Blum K. (2017). KB220Z a Pro-Dopamine Regulator Associated with the Protracted, Alleviation of Terrifying Lucid Dreams. Can We Infer Neuroplasticity-induced Changes in the Reward Circuit?. J. Reward Defic. Syndr. Addict. Sci..

[161] McLaughlin T., Han D., Nicholson J., Steinberg B., Blum K., Febo M., Braverman E., Li M., Fried L., Badgaiyan R. (2017). Improvement of long-term memory access with a pro-dopamine regulator in an elderly male: Are we targeting dopamine tone?. J. Syst. Integr. Neurosci..

[162] Steinberg B., Blum K., McLaughlin T., Lubar J., Febo M., Braverman E.R., Badgaiyan R.D. (2016). Low-Resolution Electromagnetic Tomog-raphy (LORETA) of changed Brain Function Provoked by Pro Dopamine Regulator (KB220z) in one Adult ADHD case. Open J. Clin. Med. Case Rep..

[163] Duquette L.L., Mattiace F., Blum K., Waite R.L., Boland T., McLaughlin T., Dushaj K., Febo M., Badgaiyan R.D. (2016). Neurobiology of KB220Z-Glutaminergic-Dopaminergic Optimization Complex [GDOC] as a Liquid Nano: Clinical Activation of Brain in a Highly Functional Clinician Improving Focus, Motivation and Overall Sensory Input Following Chronic Intake. Clin. Med. Rev. Case Rep..

[164] Mclaughlin T., Oscar-Berman M., Simpatico T., Giordano J., Jones S., Barh D., Downs W.B., Waite R.L., Madigan M., Dushaj K. (2013). Hypothesizing repetitive paraphilia behavior of a medication refractive Tourette’s syndrome patient having rapid clinical attenuation withKB220Z-nutrigenomic amino-acid therapy (NAAT). J. Behav. Addict..

[165] Solanki N., Abijo T., Galvao C., Darius P., Blum K., Gondré-Lewis M.C. (2020). Administration of a putative pro-dopamine regulator, a neuronutrient, mitigates alcohol intake in alcohol-preferring rats. Behav Brain Res..

[166] Blum K., Cull J.G., Chen T.J., Garcia-Swan S., Holder J.M., Wood R., Braverman E.R., Bucci L.R., Trachtenberg M.G. (1997). Clinical evidence For Effectiveness of Phencal in maintaining weight loss in an open-label, controlled, 2-year study. Curr. Ther. Res..

[167] Blum K., Modestino E.J., Lott L., Siwicki D., Baron D., Howeedy A., Badgaiyan R.D. (2018). Introducing “Precision Addiction Management (PAM^®^)” as an Adjunctive Genetic Guided Therapy for Abusable Drugs in America. Open Access J. Behav. Sci. Psychol..

[168] Steinberg B., Carey E., Modestino J.E., Mclaughlin T., Lubar J., Thanos P.K., Baron D., Blum K. (2019). Pro-Dopamine Regulation with KB220Z Improves Working Memory in an Adult with ADHD-A Case Report and Replication. Open J. Clin. Med. Case Rep..

[169] Schoenthaler S.J., Blum K., Braverman E.R., Giordano J., Thompson B., Berman M.O., Badgaiyan R.D., Madigan M.A., Dushaj K., Li M. (2015). NIDA-Drug Addiction Treatment Outcome Study (DATOS) Relapse as a Function of Spirituality/Religiosity. J. Reward Defic. Syndr..

[170] Wallace C., Burns L., Gilmour S., Hutchinson D. (2007). Substance use, psychological distress and violence among pregnant and breastfeeding Australian women. Aust. N. Z. J. Public Health.

[171] Lobmaier P., Gossop M., Waal H., Bramness J. (2010). The pharmacological treatment of opioid addiction—A clinical perspective. Eur. J. Clin. Pharmacol..

[172] Gyarmathy V.A., Giraudon I., Hedrich D., Montanari L., Guarita B., Wiessing L. (2009). Drug use and pregnancy—Challenges for public health. Eurosurveillance.

[173] Florey C.D. (1992). EUROMAC. A European concerted action: Maternal alcohol consumption and its relation to the outcome of pregnancy and child development at 18 months. Methods. Int. J. Epidemiol..

[174] Mactier H. (2011). The management of heroin misuse in pregnancy: Time for a rethink?. Arch. Dis. Child Fetal Neonatal Ed..

[175] Blum K., Hamilton M.G., Hirst M., Wallace J.E. (1978). Putative Role of Isoquinoline Alkaloids in Alcoholism: A Link to Opiates. Alcohol. Clin. Exp. Res..

[176] Blum K., Eubanks J.D., Wallace J.E., Schwertner H., Morgan W.W. (1976). Possible role of tetrahydroisoquinoline alkaloids in post alcohol intoxication states. Ann. N. Y. Acad. Sci..

[177] Blum K., Wallace J. (1974). Effects of catecholamine synthesis inhibition on ethanol-induced withdrawal symptoms in mice. J. Cereb. Blood Flow Metab..

[178] Blum K., Eubanks J.D., Wallace J.E., Hamilton H. (1976). Enhancement of Alcohol Withdrawal Convulsions in Mice by Haloperidol. Clin. Toxicol..

[179] Blum K., Wallace J.E., Schwertner H.A., Eubanks J.D. (1976). Enhancement of ethanol-induced withdrawal convulsions by blockade of 5-hydroxytryptamine receptors. J. Pharm. Pharmacol..

[180] Blum K., Eubanks J.D., Wallace J.E., Schwertner H.A. (1976). Suppression of ethanol withdrawal by dopamine. Experientia.

[181] Blum K., Wallace J.E., Schwerter H.A., Eubanks J.D. (1976). Morphine suppression of ethanol withdrawal in mice. Experientia.

[182] Blum K., Baron D., McLaughlin T., Gold M.S. (2020). Molecular neurological correlates of endorphinergic/dopaminergic mechanisms in reward circuitry linked to endorphinergic deficiency syndrome (EDS). J. Neurol. Sci..

[183] Blum K., Futterman S., Wallace J.E., Schwertner H.A. (1977). Naloxone-induced inhibition of ethanol dependence in mice. Nature.

[184] Blum K., Briggs A.H., DeLallo L. (1983). Clonidine enhancement of ethanol withdrawal in mice. Subst. Alcohol Actions Misuse.

[185] Roehrich H., Gold M.S. (1988). Clonidine. Adv. Alcohol Subst. Abus..

[186] Gold M.S., Blum K. (2021). Clonidine: The Locus Coeruleus & Noradrenergic Hyperactivity Theory for Opioid and other Drug Withdrawal from 1977 to Present. Clonidine.

[187] Noble E.P. (1994). Polymorphisms of the D2 dopamine receptor gene and alcoholism and other substance use disorders. Alcohol Alcohol Suppl..

[188] Comings D.E., Blum K. (2000). Reward deficiency syndrome: Genetic aspects of behavioral disorders. Prog. Brain Res..

[189] Noble E.P. (2003). D2 dopamine receptor gene in psychiatric and neurologic disorders and its phenotypes. Am. J. Med. Genet. Part B Neuropsychiatr. Genet..

[190] Gyollai A., Griffiths M., Barta C., Vereczkei A., Urban R., Kun B., Kökönyei G., Szekely A., Sasvari-Szekely M., Blum K. (2014). The Genetics of Problem and Pathological Gambling: A Systematic Review. Curr. Pharm. Des..

[191] Bidwell L.C., Karoly H.C., Thayer R.E., Claus E.D., Bryan A.D., Weiland B.J., YorkWilliams S., Hutchison K.E. (2018). *DRD2*promoter methylation and measures of alcohol reward: Functional activation of reward circuits and clinical severity. Addict. Biol..

[192] Blum K., Chen A.L., Thanos P.K., Febo M., Demetrovics Z., Dushaj K., Kovoor A., Baron D., Smith D.E., Lll A.K.R. (2018). Genetic addiction risk score GARS trade a predictor of vulnerability to opioid dependence. Front. Biosci..

[193] Kótyuk E., Urbán R., Hende B., Richman M., Magi A., Király O., Barta C., Griffiths M.D., Potenza M.N., Badgaiyan R.D. (2022). Development and validation of the Reward Deficiency Syndrome Questionnaire (RDSQ-29). J. Psychopharmacol..

[194] Blum K., Bowirrat A., Baron D., Lott L., Ponce J.V., Brewer R., Siwicki D., Boyett B., Gondre-Lewis M.C., Smith D.E. (2020). Biotechnical development of genetic addiction risk score (GARS) and selective evidence for inclusion of polymorphic allelic risk in substance use disorder (SUD). J. Syst. Integr. Neurosci..

[195] Gupta A., Bowirrat A., Gomez L.L., Baron D., Elman I., Giordano J., Jalali R., Badgaiyan R.D., Modestino E.J., Gold M.S. (2022). Hypothesizing in the Face of the Opioid Crisis Coupling Genetic Addiction Risk Severity (GARS) Testing with Electrotherapeutic Nonopioid Modalities Such as H-Wave Could Attenuate Both Pain and Hedonic Addictive Behaviors. Int. J. Environ. Res. Public Health.

[196] Blum K., Morgan J., Cadet J.L., Baron D., Carney P.R., Khalsa J., Badgaiyan R.D., Gold M.S. (2021). Psychoactive Drugs Like Cannabis -Induce Hypodopaminergic Anhedonia and Neuropsychological Dysfunction in Humans: Putative Induction of Dopamine Homeo-stasis via Coupling of Genetic Addiction Risk Severity (GARS) testing and Precision Pro-dopamine Regulation (KB220). Neurology.

[197] Blum K., Siwicki D., Baron D., Modestino E., Badgaiyan R.D. (2018). The benefits of genetic addiction risk score (GARS^®^) and pro-dopamine regulation in combating suicide in the American Indian population. J. Syst. Integr. Neurosci..

[198] Blum K., Gold M.S., Llanos-Gomez L., Jalali R., Thanos P.K., Bowirrat A., Downs W.B., Bagchi D., Braverman E.R., Baron D. (2021). Hypothesizing Nutrigenomic-Based Precision Anti-Obesity Treatment and Prophylaxis: Should We Be Targeting Sarcopenia Induced Brain Dysfunction?. Int. J. Environ. Res. Public Health.

[199] Blum K., Modestino E.J., Gondre-Lewis M., Chapman E.J., Neary J., Siwicki D., Baron D., Hauser M., Smith D.E., Roy A.K. (2018). The Benefits of Genetic Addiction Risk Score (GARS™) Testing in Substance Use Disorder (SUD). Int. J. Genom. Data. Min..

[200] Blum K., Steinberg B., Gondré-Lewis M.C., Baron D., Modestino E.J., Badgaiyan R.D., Downs B.W., Bagchi D., Brewer R., McLaughlin T. (2021). A Review of DNA Risk Alleles to Determine Epigenetic Repair of mRNA Expression to Prove Therapeutic Effectiveness in Reward Deficiency Syndrome (RDS): Embracing “Precision Behavioral Management”. Psychol. Res. Behav. Manag..

[201] Blum K., Gold M., Modestino E.J., Baron D., Boyett B., Siwicki D., Lott L., Podesta A., Roy A.K., Hauser M. (2018). Would induction of dopamine homeostasis via coupling genetic addiction risk score (GARS^®^) and pro-dopamine regulation benefit benzodiazepine use disorder (BUD). J. Syst. Integr. Neurosci..

[202] Moran M., Blum K., Ponce J.V., Lott L., Gondré–Lewis M.C., Badgaiyan S., Brewer R., Downs B.W., Fynman P., Weingarten A. (2021). High Genetic Addiction Risk Score (GARS) in Chronically Prescribed Severe Chronic Opioid Probands Attending Multi-pain Clinics: An Open Clinical Pilot Trial. Mol. Neurobiol..

[203] Blum K., Oscar-Berman M., Demetrovics Z., Barh D., Gold M.S. (2014). Genetic Addiction Risk Score (GARS): Molecular Neurogenetic Evidence for Predisposition to Reward Deficiency Syndrome (RDS). Mol. Neurobiol..

[204] Fried L., Modestino E.J., Siwicki D., Lott L., Thanos P.K., Baron D., Badgaiyan R.D., Ponce J.V., Giordano J., Downs W.B. (2020). Hypodopaminergia and “Precision Behavioral Management” (PBM): It is a Generational Family Affair. Curr. Pharm. Biotechnol..

[205] Blum K., Baron D., Lott L., Ponce J.V., Siwicki D., Boyett B., Steinberg B., Modestino E.J., Fried L., Hauser M. (2020). In Search of Reward Deficiency Syndrome (RDS)-Free Controls: The “Holy Grail” in Genetic Addiction Risk Testing. Curr. Psychopharmacol..

[206] Blum K., McLaughlin T., Modestino E.J., Baron D., Bowirrat A., Brewer R., Steinberg B., Roy A.K., Febo M., Badgaiyan R.D. (2021). Epigenetic Repair of Terrifying Lucid Dreams by Enhanced Brain Reward Functional Connectivity and Induction of Dopaminergic Homeo—Static Signaling. Curr. Psychopharmacol..

[207] Blum K., Modestino E.J., Neary J., Gondré-Lewis M.C., Siwicki D., Moran M., Hauser M., Braverman E.R., Baron D., Steinberg B. (2018). Promoting Precision Addiction Management (PAM) to Combat the Global Opioid Crisis. Biomed. J. Sci. Tech. Res..

[208] Blum K., Badgaiyan R.D., Agan G., Fratantonio J., Simpatico T., Febo M., Haberstick B.C., Smolen A., Gold M.S. (2015). Molecular Genetic Testing in Reward Deficiency Syndrome (RDS): Facts and Fiction. J. Reward Defic. Syndr..

[209] Blum K., Lott L., Siwicki D., Fried L., Hauser M., Simpatico T., Baron D., Howeedy A., Badgaiyan R.D. (2018). Genetic Addiction Risk Score (GARS™) as a Predictor of Substance Use Disorder: Identifying Predisposition Not Diagnosis. Curr. Trends Med. Diagn. Methods.

[210] Blum K., Gondré-Lewis M.C., Baron D., Thanos P.K., Braverman E.R., Neary J., Elman I., Badgaiyan R.D. (2018). Introducing Precision Addiction Management of Reward Deficiency Syndrome, the Construct That Underpins All Addictive Behaviors. Front. Psychiatry.

[211] Blum K., Oscar-Berman M., Blum S.H., Madigan M.A., Waite R.L., McLaughlin T., Barh D.O. (2014). Can Genetic Testing Coupled with Enhanced Dopaminergic Activation Reduce Recidivism Rates in the Workers Compensation Legacy Cases?. J. Alcohol. Drug Depend..

[212] Blum K., Bowirrat A., Lewis M.C., Simpatico T.A., Ceccanti M., Steinberg B., Modestino E.J., Thanos P.K., Baron D., McLaughlin T. (2021). Exploration of Epigenetic State Hyperdopaminergia (Surfeit) and Genetic Trait Hypodopaminergia (Deficit) during Adolescent Brain Development. Curr. Psychopharmacol..

[213] Talwar P., Silla Y., Grover S., Gupta M., Agarwal R., Kushwaha S., Kukreti R. (2014). Genomic convergence and network analysis approach to identify candidate genes in Alzheimer’s disease. BMC Genom..

